# Pregnancy planning in women with rheumatic diseases: an integrated framework for risk stratification and multidisciplinary management

**DOI:** 10.1007/s00296-026-06117-0

**Published:** 2026-04-24

**Authors:** Dinara Yerlanova, Dinara Makhanbetkulova, Umida Khojakulova, Yuliya Fedorchenko, Olena Zimba, Ahmet Usen

**Affiliations:** 1 Department of Chemical Disciplines, Biology and Biochemistry, South Kazakhstan Medical Academy, Shymkent, Kazakhstan; 2 Department of Nursing, Asfendiyarov Kazakh National Medical University, Almaty, Kazakhstan; 3 Department of Emergency Medicine and Nursing, South Kazakhstan Medical Academy, Shymkent, Kazakhstan; 4 Department of Pathophysiology, Ivano-Frankivsk National Medical University, Ivano-Frankivs’k, Ukraine; 5 Department of Rheumatology, Immunology and Internal Medicine, University Hospital in Kraków, Kraków, Poland; 6 National Institute of Geriatrics, Rheumatology and Rehabilitation, Warsaw, Poland; 7 Department of Internal Medicine N2, Danylo Halytsky Lviv National Medical University, Lviv, Ukraine; 8 Department of Physical Medicine and Rehabilitation, Faculty of Medicine, Medipol University, Istanbul, Türkiye

**Keywords:** Rheumatic Diseases, Fertility Preservation, Preconception Care, Autoimmune Diseases, Pregnancy Outcome

## Abstract

Pregnancy planning in patients with rheumatic diseases (RD) is a multifaceted public health issue that includes complex strategies targeting underlying disease activity and reproductive potential. Patients with autoimmune diseases, including those with systemic lupus erythematosus (SLE), rheumatoid arthritis (RA), Sjögren syndrome, systemic sclerosis (SSc), and spondyloarthritis, present with heightened risks of adverse maternal and fetal outcomes, including miscarriage, fetal growth restriction, preeclampsia, preterm birth, and increased utilization of assisted reproductive technologies. Available evidence suggests that conception during sustained disease remission, alongside tailored drug therapies, enhances maternal-fetal outcomes. Critical determinants of obstetric prognosis include disease-specific activity indices, serologic markers, organ involvement, and prior gonadotoxic exposures which compromise ovarian reserve. Structured preconception counseling that integrates contraception strategies, fertility preservation, ovarian reserve assessment, and multidisciplinary follow-up is essential to mitigate these risks. Current management paradigms advocate the continuation of pregnancy-compatible disease-modifying antirheumatic drugs (DMARDs), while strictly avoiding teratogenic therapies. Despite these advances, substantial unmet needs exist in early risk stratification, patient education, and systematic integration of reproductive health counselling into routine RD management. This review synthesizes contemporary evidence, delineates existing gaps, and provides a strategic framework for optimizing pregnancy planning and reproductive outcomes in women with RDs. The review addresses the key domains, including preconception risk stratification, disease-specific considerations, fertility assessment, ovarian reserve evaluation, and optimization of drug therapies. A multidisciplinary, treat-to-target approach is essential to improve pregnancy outcomes and long-term maternal health in this high-risk population.

## Introduction

**Fig. 1:**
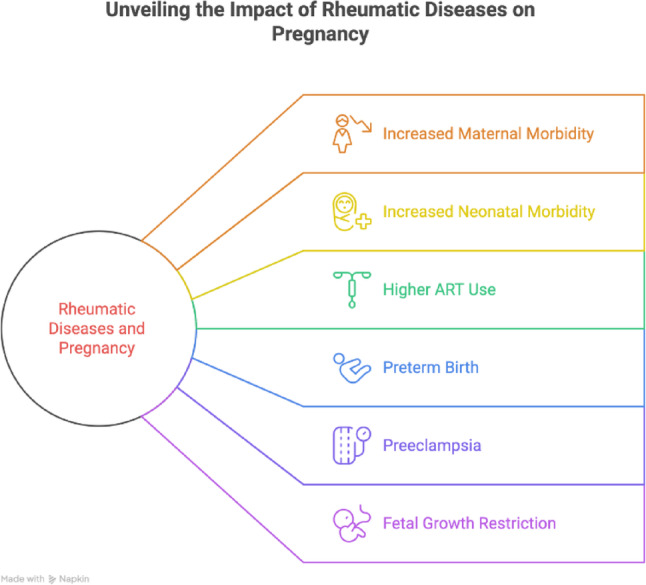
Major Maternal and Fetal Risks Associated with Pregnancy in Women with Rheumatic Diseases

Rheumatic diseases (RD) are chronic immune-mediated conditions that predominantly affect women during their reproductive years, distinguishing pregnancy planning as a public health issue [1, 2]. Accumulating evidence suggests that pregnancies in RD patients are associated with increased risks of maternal and fetal adverse outcomes, necessitating structured preconception counselling and multidisciplinary management [3–6]. In a Japanese multicenter study of 69,810 deliveries, pregnancies in women with autoimmune diseases, including systemic lupus erythematosus (SLE) and rheumatoid arthritis (RA), demonstrated high-risk profiles, with fewer than 50% planned conceptions and frequent use of assisted reproductive technologies in RA (23%) and SLE (11.4%) [7]. Preterm birth was registered in 39.4% of SLE and 27.5% of RA patients, preeclampsia in 15% of SLE cases, and fetal growth restriction in 13% of SLE cases [7]. Systematic reviews point to high risk of miscarriage in women with SLE and Sjögren syndrome [4, 8]. Taken together, available evidence underscores the multidimensional effects of RDs on maternal and fetal outcomes (Fig. 1).

Recent high-level evidence emphasizes that coordinated preconception counselling and tight disease control before conception substantially improve maternal and fetal outcomes, shifting pregnancy management from risk avoidance toward proactive planning within specialized multidisciplinary frameworks [9].

Pregnancy has been discouraged in women with active SLE, particularly in the presence of lupus nephritis, antiphospholipid antibodies, or immunosuppressive therapy [10, 11]. Favorable pregnancy outcomes are expected when conception occurs during sustained remission (≥ 6 months) with controlled disease activity and optimized therapies [10, 12].

The aim of this review is to delineate unmet needs in pregnancy planning for women with RDs, encompassing contraception counselling, infection screening, fertility preservation, ovarian reserve assessment, safe drug therapies, and structured multidisciplinary follow-up.

### Search strategy

A comprehensive literature search was performed via Medline/PubMed, Scopus, and Directory of Open Access Journals **(**DOAJ) to identify articles on pregnancy planning, reproductive risk, fertility preservation, and drug therapies in women with RDs. Eligible documents included observational studies, registry analyses, systematic reviews, and practice guidelines published up to March 1, 2026.

Search terms combined controlled vocabulary (MeSH/Emtree) and free-text keywords using Boolean operators:

(“rheumatic disease” OR “autoimmune rheumatic disease” OR “rheumatoid arthritis” OR “systemic lupus erythematosus” OR “antiphospholipid syndrome” OR “Sjögren’s syndrome” OR “systemic sclerosis” OR “spondyloarthritis” OR “psoriatic arthritis” OR “ankylosing spondylitis”)

AND.

(“pregnancy planning” OR “preconception counselling” OR “fertility” OR “ovarian reserve” OR “assisted reproduction” OR “contraception”)

AND.

(“pregnancy outcome” OR “preeclampsia” OR “preterm birth” OR “miscarriage” OR “fetal growth restriction” OR “congenital heart block”)

AND.

(“disease activity” OR “risk stratification” OR “multidisciplinary management” OR “treat-to-target” OR “pharmacologic management” OR “DMARD” OR “biologic therapy” OR “teratogenicity”).

Only English articles were analyzed. Searches were in line with widely publicized relevant recommendations [13].

### Preconception risk stratification and multidisciplinary framework

RD activity at conception represents the principal modifiable determinant of obstetric outcome [14, 15]. A preconception SLEDAI ≥ 4, hypocomplementemia, and elevated anti-dsDNA titers predict maternal flares and adverse obstetric outcomes in lupus, while active lupus nephritis markedly increases early pregnancy renal relapse risk [12]. Likewise, higher rheumatoid activity correlates with prolonged time to conception and increased obstetric complications [16]. Registry-based analyses of Swedish and Danish cohorts (*n* = 3,047 pregnancies) demonstrate increased risks of preeclampsia in RA (adjusted OR [aOR] 1.27), axial spondyloarthritis (aOR 1.17), and psoriatic arthritis (aOR 1.85) [17]. A systematic review of studies with over 50 million participants confirmed that RA was associated with heightened risks of cesarean delivery (OR 1.39), preeclampsia (OR 1.48), preterm birth (OR 1.58), small-for-gestational-age neonates (OR 1.49), and stillbirth (OR 1.38) in RA [18].

### Disease-specific considerations

#### Systemic Lupus Erythematosus and Antiphospholipid syndrome

In SLE, live birth rates exceeding 80% are achievable with high rates of preconception counselling (86.9%) and hydroxychloroquine (HCQ) use (63.7%) [19]. Adverse outcomes are still frequent (41.4%), particularly in women with active lupus, lupus nephritis, hypocomplementemia, and antiphospholipid syndrome (APS) [19]. Anti-Ro/SSA antibodies confer a 17.9% risk of congenital heart block, supporting the need for targeted fetal echocardiographic surveillance [20].

The European Alliance of Associations for Rheumatology (EULAR) framework for SLE/APS pregnancy planning emphasizes early counselling, individualized risk stratification (organ damage, renal function, antibody profile), and low-dose acetylsalicylic acid and heparin therapies [21, 22]. The key measures include fertility assessment, assisted reproduction, contraception, and menopause and malignancy surveillance in women with RDs [22].

Fertility in SLE patients can be impaired by active disease, renal dysfunction, menstrual irregularities, or cyclophosphamide-induced ovarian insufficiency, which is age- and dose-dependent [23]. Gonadotropin-releasing hormone analogues administered before or with cyclophosphamide may reduce premature ovarian failure and improve reproductive outcomes [24].

HCQ is recommended preconceptionally and throughout gestation [25]. Corticosteroids, azathioprine, and calcineurin inhibitors can be used in active lupus [26–28]. Cyclophosphamide, mycophenolate mofetil, leflunomide, and methotrexate are contraindicated during pregnancy, while biologics such as belimumab are reserved for refractory cases [29, 30]. In APS, combined low-dose aspirin and heparin therapies are mandatory, with adjunctive vitamin D and calcium supplementation to preserve maternal bone health [31, 32]. HCQ therapy is recommended throughout pregnancy to reduce flare risk and lower the risk of congenital heart block [33, 34].

### Rheumatoid arthritis

Although RA activity often improves during gestation, postpartum flares occur in up to 74% of patients [35]. High rheumatoid activity is associated with preterm birth and fetal growth restriction, reinforcing the importance of DAS28–based monitoring [16, 36]. Optimal pregnancy outcomes are expected when conception is planned in women with low or inactive disease, with continuation of compatible medications to prevent flares [37]. EULAR and the American College of Rheumatology (ACR) advocate for the continuation of pregnancy-compatible DMARDs to maintain remission [38, 39].

Preconception counselling for women with RA requires a structured assessment of disease course, RA-specific autoantibodies, and DAS28 [40]. Laboratory tests should cover hematologic, metabolic, renal, hepatic, thyroid, and coagulation profiles [40, 41]. Comorbidity assessment, reproductive history, vaccination status, and supplementation with folic acid and vitamin D are essential to ensure safe conception and favorable pregnancy outcomes [41].

### Sjögren syndrome

Pregnancies in Sjögren syndrome (SS) are complicated due to transplacental passage of anti-SSA/SSB antibodies [42–43]. There is increasing risks of congenital heart block (CHB), miscarriage, preterm birth and higher admissions to neonatal intensive care unit (NICU) [44, 45].

CHB in structurally normal fetuses is mainly associated with transplacental anti-SSA/SSB antibodies and arises at 16–24 weeks [46]. In four pregnancies—including one high-risk case with SS—immunoadsorption at 19–25 weeks lowered SSA titers by 47–80% and resolved hydrops, though complete CHB remained irreversible, requiring pacing [46].

Preconception antibody screening, counselling on recurrence risk, and structured fetal cardiac monitoring are essential [47].

In primary Sjögren disease, placentas show elevated macrophage infiltration, with unchanged lymphocyte populations and absent plasmacytoid dendritic cells, indicating that macrophage-driven interferon activation may underlie the increased risk of adverse pregnancy outcomes [48].

### Systemic sclerosis

Pregnancies in patients with systemic sclerosis (SSc) carry significantly elevated risks of miscarriage (OR 1.6), intrauterine growth restriction (OR 3.2), preterm birth (OR 2.4), and gestational hypertension (OR 2.8) [49]. In a cohort study on 154 pregnancies (2008–2019), SSc was associated with substantially higher rates of adverse pregnancy outcomes compared with healthy controls (61% vs. 10%) [50]. SSc pregnancies resulted in first-trimester miscarriages (15%), preeclampsia (12%), small-for-gestational-age neonates (21.2%), and preterm delivery (24.2%) [50]. Nationwide Swedish data indicate increased preeclampsia (RR 3.8) and preterm birth (RR 3.3) [51].

Consistent with these findings, among 58 SSc pregnancies, 53 (91.4%) resulted in live births and 14 (26.4%) experienced adverse outcomes, including placental dysfunction, small-for-gestational-age infants, preeclampsia, preterm birth, low birthweight, and severe postpartum hemorrhage [52]. Maternal disease worsened in 23 cases (39.7%), underscoring the critical need for specialized multidisciplinary management to optimize both maternal and fetal outcomes [52].

Preconception counselling in SSc should address teratogenic drug discontinuation (methotrexate, mycophenolate mofetil), cautious corticosteroid dosing (< 15 mg prednisolone), and individualized risk assessment for renal crisis and cardiopulmonary involvement [53]. Most women with SSc need satisfactory preconception counselling on contraceptive use [54].

### Spondyloarthritis

In ankylosing spondylitis (AS) and psoriatic arthritis (PsA), active disease is associated with increased risks of preterm birth (aRR up to 1.8), oligohydramnios (aRR 3.15–3.79), cesarean delivery (aRR 1.57–1.63), and small-for-gestational-age infants (aRR up to 7.04) [55, 56]. High disease activity and second-trimester glucocorticoid exposure further amplify the risk of adverse outcomes [56].

### Ovarian reserve and fertility preservation

Impaired fertility is a big issue for pregnancy planning. Anti-Müllerian hormone (AMH), a validated marker of ovarian reserve [57–60]. This marker is significantly decreased in RA, SLE, SSc, Behçet disease, and SpA, compared with healthy controls [60]. In SpA, HLA-B27 positivity is associated with further decline in AMH [60]. Systematic reviews have confirmed AMH decline in women with SLE, particularly due to cyclophosphamide exposure [58, 59]. Given the age- and dose-dependent gonadotoxicity of cyclophosphamide, fertility preservation strategies should be discussed prior to the treatment initiation [61]. In women at risk of treatment-related ovarian insufficiency, fertility preservation options—including oocyte or embryo cryopreservation and the use of gonadotropin-releasing hormone analogues during gonadotoxic therapy—should be considered early in the course of treatment [24, 62].

### Optimization of drug therapies

Safe drug therapies are integral to pregnancy planning in RDs. According to available practice guidelines, hydroxychloroquine, azathioprine, sulfasalazine, calcineurin inhibitors, and anti-TNF agents are compatible with conception and gestation, whereas methotrexate, mycophenolate, cyclophosphamide, and leflunomide require discontinuation prior to conception [34, 62].

Structured preconception counselling, including specialist referral, is advisable to ensure disease quiescence, appropriate contraception, and individualized drug therapies prior to the conception [34, 63]. Pregnancy-incompatible medications should be discontinued or substituted with safer alternatives while maintaining RD control [64]. In severe disease, continuation of anti-inflammatory therapies, including biologic DMARDs, is justified [65].

Selected conventional synthetic DMARDs and immunomodulatory agents, including azathioprine, 6-mercaptopurine, colchicine, cyclosporine, hydroxychloroquine, sulfasalazine, and tacrolimus, can be used during conception planning [62]. TNFalpha inhibitors, belimumab, rituximab, abatacept, and anakinra are acceptable during the preconception phase [66]. Medications with limited safety data, such as anifrolumab, guselkumab, risankizumab, apremilast, baricitinib, filgotinib, tofacitinib, upadacitinib, and voclosporin, should be avoided [62].

Among pregnancy-compatible agents, HCQ (≤ 400 mg/day) is the preferred antimalarial. Evidence from more than 4,700 reported exposures to this agent demonstrates no association with adverse gestational outcomes, low birth weight, or congenital anomalies, and suggests a reduced miscarriage rate compared with unexposed pregnancies [34].

Glucocorticoids are adjunctive agents for RD control. Prednisolone and methylprednisolone undergo substantial placental metabolism, resulting in limited fetal exposure and compatibility with pregnancy [63]. However, prolonged or high-dose glucocorticoid therapies may increase the risk of preterm birth and low birth weight [67]. Prednisolone at < 20 mg/day is preferred, ideally in combination with pregnancy-compatible steroid-sparing agents [39, 68].

Methotrexate should be withdrawn at least 3 months before conception [35, 63]. Sulfasalazine is safe throughout pregnancy provided folic acid supplementation (5 mg/day) is administered periconceptionally [34, 69]. Leflunomide is a non-preferred agent in women planning pregnancy [34, 70]. Cyclophosphamide and mycophenolate should also be discontinued before conception [62, 71, 72]. Low-dose aspirin from 12-week gestation reduces preeclampsia risk, and low-molecular-weight heparin is essential in APS [73].

### Fertility and reproductive risk in rheumatic disease

Reproductive health in women with RD is influenced by both intrinsic disease mechanisms and cumulative therapeutic exposures, necessitating structured preconception assessment [74]. A survey of rheumatologists and obstetricians revealed specialists’ awareness of reduced fertility, recurrent miscarriages, fetal malformations, and preeclampsia across RDs [75]. Spondyloarthritis is perceived as low-risk disease during pregnancy [75].

Despite guideline familiarity, knowledge gaps persist. In a survey of 122 rheumatologists, most reported confidence in managing contraception and breastfeeding in women with RD and awareness of the BSR 2023 and ACR 2020 recommendations [76]. However, substantial uncertainty remained regarding oocyte preservation (63% reported low confidence) and assisted reproductive technologies (65.9%) [76]. Nearly all respondents noted patient concerns about pregnancy planning (92%) and premature discontinuation of medications following conception (50.8%), with inadequate treatment and follow-up identified as major contributors to adverse pregnancy outcomes (91.7%) [76].

RA is associated with increased rates of infertility, endometriosis, and premature ovarian insufficiency, with potential links to polycystic ovary syndrome and pregnancy loss [77]. Chronic disease burden and psychosocial stress may delay family planning, reinforcing the need for early reproductive counselling and individualized preconception strategies in women with RA [77].

Comorbidities such as renal insufficiency and prior cyclophosphamide therapy markedly impair ovarian reserve, with risk escalating according to maternal age and cumulative drug dose [61, 78, 79]. Pelvic floor dysfunction has also been reported in women with RDs, suggesting that pelvic floor assessment and rehabilitation strategies may represent supportive components of multidisciplinary reproductive care [80, 81].

### Contraception counselling

**Fig. 2:**
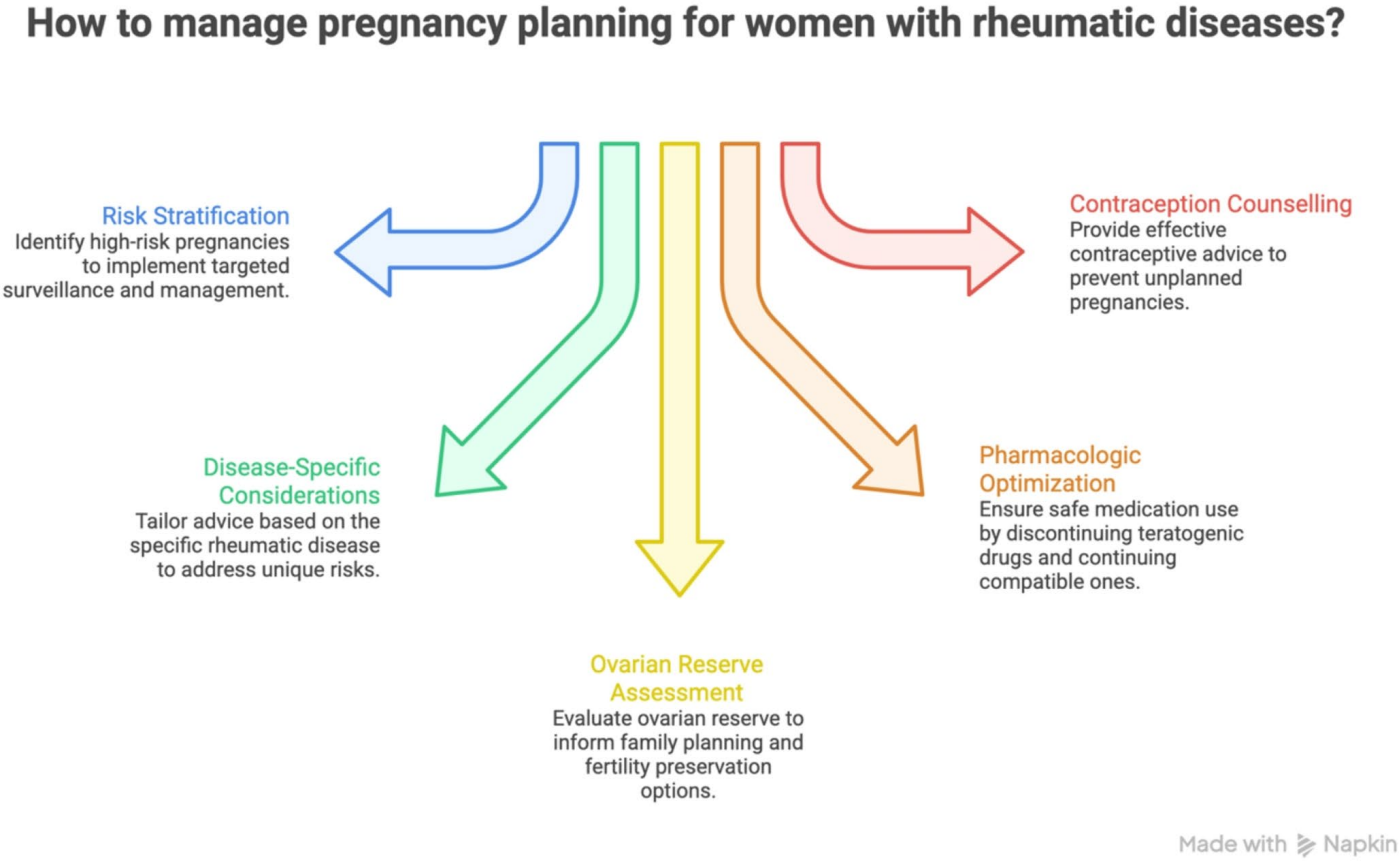
Core domains of pregnancy planning in women with rheumatic diseases

Global declines in fertility and persistent unmet contraceptive needs highlight the critical role of effective family planning, particularly for women at increased reproductive risk [82]. Contraceptive counselling is important in women on teratogenic drug therapies [83]. In this context, long-acting reversible contraceptive methods, including intrauterine devices and implants, are often preferred in women receiving teratogenic therapies due to their high efficacy and reduced dependence on user adherence [39, 83]. RA patients may use any effective contraceptive method, provided thrombotic risk and antibody status are considered [83, 84]. Early family planning discussions reduce unplanned pregnancy rates and enable transition to pregnancy-compatible regimens. A structured, multidisciplinary approach to pregnancy planning in women with rheumatic diseases is summarized in Fig. 2, highlighting the core domains of preconception management.

## Conclusion

Pregnancy planning in women with RDs requires proactive and individualized care. Disease remission at conception, ovarian reserve assessment, teratogenic drug withdrawal, and continuation of compatible immunomodulatory therapies mitigate maternal risk. Despite therapeutic advances, substantial unmet needs exist in fertility counselling, contraceptive guidance, and structured preconception assessment. Integrating standardized, evidence-based counselling frameworks into routine rheumatologic care is essential to optimize reproductive outcomes and long-term maternal health in RDs.
